# Overview of Current International Recommendations for Echocardiography Exams During the Covid-19 Pandemic and Its Local Implementation in Austria

**DOI:** 10.3389/fcvm.2021.623076

**Published:** 2021-02-10

**Authors:** Michael Lichtenauer, Erika Prinz, Christina Granitz, Bernhard Wernly, Kristen Kopp, Apollonia Daburger, Uta C. Hoppe

**Affiliations:** Department of Internal Medicine II, Division of Cardiology, Paracelsus Medical University of Salzburg, Salzburg, Austria

**Keywords:** COVID-19, echocardiography, protective gear, cardiovascular imaging, SARS–CoV−2

## Abstract

Since its first appearance in December 2019, the novel Coronavirus SARS-CoV-2 (Severe acute respiratory syndrome coronavirus 2) has spread throughout the world at rapid pace causing the *coronavirus disease 2019* (Covid-19). Originating in the Chinese province Hubei, more than 91.8 million people globally have now been infected with the coronavirus and more than 1.966.000 patients have died thus far from Covid-19 (as of January 13th 2021). The virus spreads primarily by droplet infection as well as via aerosols during close physical contact. Particularly in medical examinations with close physical contact between examiner and patient, like echocardiography, the risk of contracting the virus is increased. Therefore, the use of personal protective equipment is recommended for the protection of patients and medical personnel alike. In this article, the current recommendations of international professional associations on the use of personal protective equipment and their local implementation are presented.

## Introduction

Close physical contact with patients suffering from *coronavirus disease 19* (Covid-19), may result in a significantly increased risk of transmission by droplet infection or via aerosols (1–3). Transthoracic echocardiography (TTE) examinations involve close patient contact over a long period of time (i.e., estimated 15–30 min). In addition to the exam length, transesophageal echocardiography (TEE) examinations in particular can result in aerosol formation. Although hard data on the extent of aerosol formation in TEE are lacking (4), some mathematical models have been proposed to explain virus transmission by aerosols even in patients with mild or asymptomatic Covid-19 (5). Special protection of patients and especially of medical staff is therefore necessary during TEE and TTE examinations. The procedure described here is based on current guidelines of the American Society of Echocardiography (6), the British Society of Echocardiography (7), the Italian Society of Echocardiography (8), the Japanese Society of Echocardiography (9), the Cardiological Society of India (10), collection of experience reports and recommendations of the European Society of Cardiology (ESC) (11, 12), as well as the recommendations of the local crisis management team of the Salzburg State hospitals.

First, the different collectives of patients must be distinguished, defined as patients with proven Covid-19 disease, patients with negative testing for SARS-CoV-2 infection, patients suspected with infection or those in which SARS-CoV-2 infection has not been excluded, as this has a decisive influence on the indication for imaging studies and on respective protective measures.

## Indications

A strict indication is of primary importance within the context of a pandemic. This applies to standard cardiological examinations in cardiac patients infected with Covid-19 as well as in non-cardiac patients with Covid-19 to evaluate possible Covid-19-associated cardiac involvement (13). Only examinations that are clearly necessary for diagnosis and that have a further therapeutic consequence should be performed in patients with suspected or confirmed Covid-19 infection, this is also of utmost importance in TEE studies with increased risk of aerosol generation (10, 14). Instead of stress echocardiography, alternative forms for testing should be considered (9).

## Implementation

**Figure 1:**
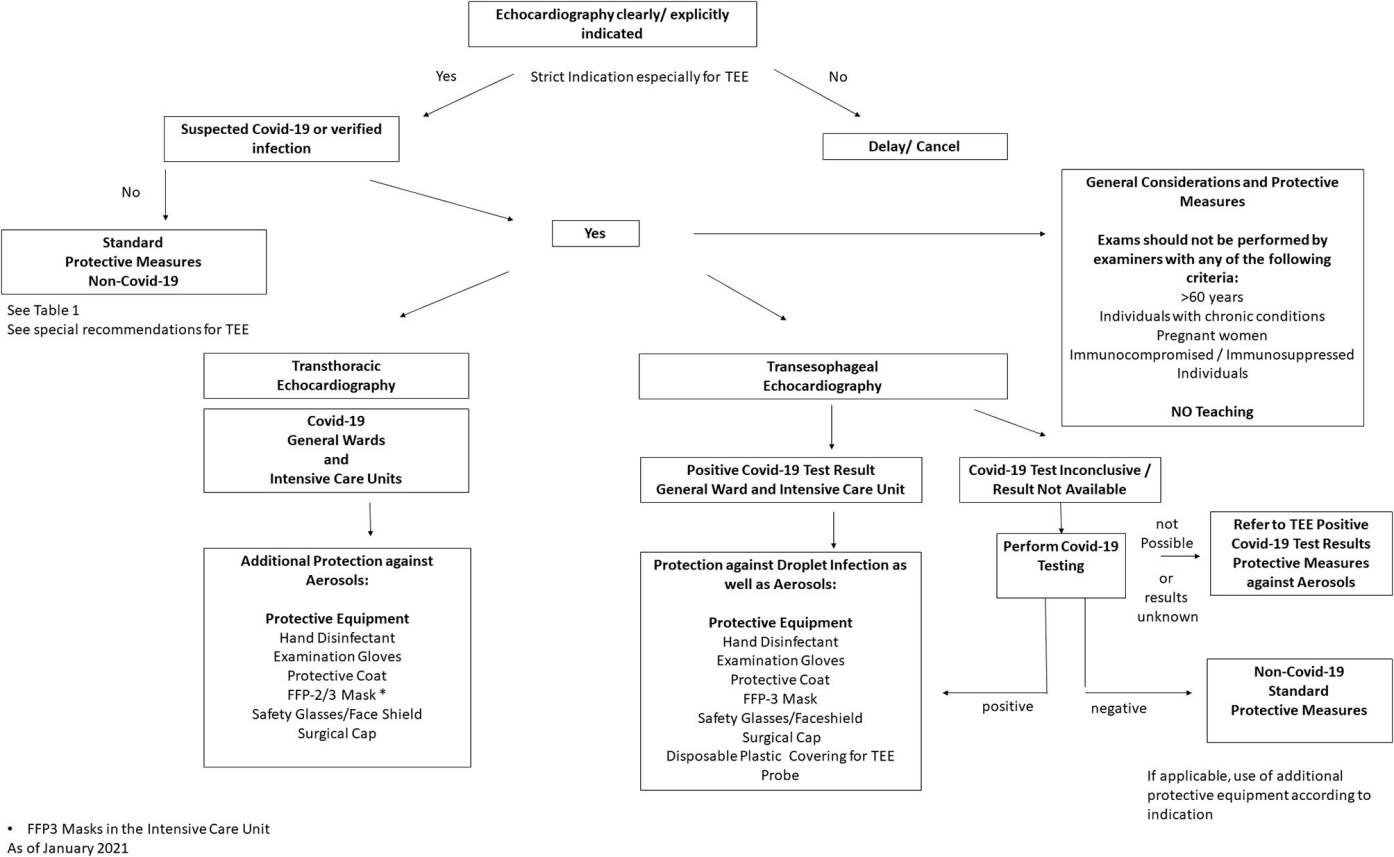
Recommended local procedure for Echocardiographic examinations during the SARS-CoV-2 pandemic [adapted from the recommendations of the American Society of Echocardiography (6)]].

Prior to performing an echocardiography examination during a pandemic, the patient's infection status should be determined in order to assess examination risk constellation. Depending on the status, appropriate protective equipment should be selected [see Section Use of Personal Protective Equipment (PPEs), Table 1, Figure 1]. Testing for SARS-CoV-2 would be desirable, especially before a TEE examination, however, in an outpatient setting this is not always feasible (no test available, or test result pending), therefore use of extensive personal protective equipment is recommended in this situation (see Table 1, “Suspected Covid-19 infection”) (15). Patients with suspected or confirmed Covid-19 infection should be examined with a mobile echocardiography device if possible to avoid virus spread by transport. This is recommended especially in designated local Covid-19 wards and Covid-19 intensive care units. The examinations should only be performed by experienced personnel to keep examination time to a minimum. Examiners > age 60, pregnant women, persons with chronic conditions (i.e., hypertension, diabetes mellitus, adipositas, COPD, and pulmonary diseases) or immunosuppressed/immunocompromised individuals should avoid contact with patients with suspected Covid-19 and those with confirmed infections. Teaching or device training should not take place when examining patients with Covid-19. Authors also recommend to use limited echocardiography protocols focusing only on the most important cardiac views in order to further reduce scan time (16). During the current pandemic, it is recommend that internet-based training and education (online lectures, webinars, and use of simulators) should replace bedside training (17, 18). In the case of suspected Covid-19 infection and where test results are pending, examinations should be delayed with the exception of urgent cases. If possible, only one examiner should perform the echocardiography on one patient per room; several examiners in the same space should be avoided (11). It is also recommended that the patient should be ideally positioned, lying on their left side facing away from the examiner. A drape covering the patient should be used to reduce physical contact if only standard protective equipment for the examiner is available. Where available, transparent drapes should be used to cover the echocardiography machine (16). The preferred examination position by the individual examiner should be maintained to ensure that the quality of the test is not compromised, as this would potentially result in re-imaging and longer examination time. The advantages and disadvantages of performing an exam must be weighed carefully. If possible, only loop recordings should be captured directly on the device and measurements and findings should be made subsequently on the computer.

**Table 1:** Personal protective equipment for echocardiography in Covid-19.

	**Hand disinfection**	**Gloves**	**Protective coat**	**Surgical mask**	**FFP 2/3 mask**	**Protective glasses/face shield**	**Surgical cap**
**TTE**	X	X	*	X	FFP 2 mask, if available		
Standard procedure Non-Covid 19 patients							
**TTE**	X	X	X		X	X	X
Covid 19 patients General ward, Intensive care unit (suspected and diagnosed infection)					(FFP-3 in Intensive Care Unit)		
**TEE**	X	X	X		X	X	X
Covid 19 patients General ward, Intensive care unit (suspected and diagnosed infection)					(FFP-3)		
**TEE**	X	X	X		X	X	X
No suspicion of Covid 19 but test result not available or inconclusive (delay TEE preferable)					(FFP-2)		
**TEE**	X	X	If needed	X FFP 2 mask, if available	If needed	If needed	If needed
Negative Covid 19 test result (increase protective measures if indicated)							

*Personal protection recommendations during echocardiographic examinations [adapted from the American Society of Echocardiography, the British Society of Echocardiography, the European Association of Cardiovascular Imaging recommendations and the Japanese Society of Echocardiography (6, 7, 9, 12)]*.

* *Use a protective drape to cover the patient if the examination position is to the right of the patient*.

Where available, handheld echocardiography devices, such as tablet-based systems, to further reduce scan time and limit the risk of exposure of personnel could be used as alternatives to conventional echocardiography, at least for screening examinations (19, 20).

After use, the device, probes and examination table should be thoroughly cleansed using disinfection towels. TEE probes should be cleaned, disinfected and sterilized according to the manufacturer's instructions. Additionally, the use of virucidal disinfectants for probe reprocessing and the use of a disposable protective covering for the TEE probe are recommended for hygienic reasons [see recommendations proposed by Jain (21)]. As a substitute to intraoperative TEE, epicardial echocardiography using a sterile sleeve has been proposed by Senniappan et al. (22). Also during cleaning procedures, personnel should wear PPE (8).

## Use of Personal Protective Equipment (PPEs)

When performing TTE or TEE in patients with suspected Covid-19 or confirmed infection, comprehensive protective equipment should be used by the examiner, consisting of examination gloves, a protective coat, a FFP-2/3 mask, safety glasses/goggles or face shield, and a surgical cap (17, 23). Local hospital recommendations and SOPs regarding correct use of personal protective equipment should be followed.

Use of comprehensive personal protection equipment (PPE) is also recommended when testing patients without proven SARS-CoV-2 infection but who have not been tested. After thorough hand disinfection, this includes use of examination gloves and a surgical mask; see Table 1, Figure 1). A new standard requirement is for patients to also wear a facemask in order to reduce patient-physician transmission risk. In addition, regular and thorough hand disinfection is recommended for both the examiner and the patient, as this is an essential element to prevent spread of the disease (24).

Special care with respect to PPEs should also be given during TEE examinations, as higher aerosol formation is to be expected (3). In the case of positive Covid-19 detection, the use of examination gloves, a protective coat, an FFP-3 mask, safety glasses/goggles or face shield and a surgical cap are recommended. If the Covid-19 test results are inconclusive, a swab PCR test should be taken before TEE examination if possible, with the exception of urgent indications. It is generally preferable to obtain results of Covid-19 tests prior to examination to save personal protective equipment resources. In the case of negative Covid-19 results or in those patients hospitalized for longer period of time without proven infection or symptoms, then standard protective measures, such as face mask and gloves may be applied (see Table and Figure). However, if a patient shows any clinical signs or laboratory signs of florid infection (i.e., fever, elevated CRP, coughing, and loss of taste), extensive personal protective equipment is recommended due to increased aerosol exposure during TEE examinations and possible false negative results. In case of unclear Covid-19 findings and urgent indication for TEE, extensive protective equipment should also be used (Table 1).

## Use of PPEs in a Pediatric Setting

The American Society of Echocardiography and the Working Group on Congenital Heart Disease of the Italian Society of Cardiology have offered specific Covid-19 examination recommendations for children and infants (25, 26). While children as an entire group appear to be at lower risk of severe infection when compared to adults, certain sub-groups of children may be more susceptible to severe disease courses and have the need for frequent examinations by means of echocardiography, e.g., children with congenital heart disease. Even though higher case-fatality rates in patients with cardiovascular diseases were initially assumed, most patients with congenital heart disease experience mild COVID-19 symptoms, though data on children remain scarce (27). Most children who are infected with SARs-CoV-2 have mild symptoms or are asymptomatic, which creates a special challenge to protect healthcare staff from exposure. TTE and TEE should only be performed if they are expected to provide clinical benefit. Given the higher risk of transmission in asymptomatic children, most centers are performing SARs-CoV-2 testing in all new pediatric admissions. If possible, imaging should be performed and images saved by a single experienced staff member and retrieved at a later time for evaluation. Prolonged scanning should be avoided. In infants and children in whom Covid-19 has not been ruled out, infection should be assumed and appropriate PPEs as well as meticulous and frequent handwashing are required. One single caregiver should accompany the child during the exam to facilitate the cooperation of an active child and should be fitted with a mask as well. Protective coverings on devices and disinfection should be done per standard protocols.

In patients with documented negative Covid-19 testing within 72 h arriving for examination risk of infection is low and standard gloves, face mask and eye protection is recommended. In patients with known Covid-19 infection or in which infection with SARS-CoV-2 cannot be ruled out, strict protocols for PPE use must be followed. If possible, staff members with risk factors (> 60 years, chronic illness, immunocompromised and pregnancy) should not perform echocardiography exams. As a general rule, children below the age of six are exempt of wearing a face mask, whereas wearing a mask in older children should be mandatory during examinations.

## Conclusion

In summary, in the context of the SARS-CoV-2 pandemic, a concise indication warranting echocardiography examination is essential to minimize transmission risk and limit use of personal protective equipment resources. It is important to emphasize that necessary echocardiographic examinations should not be postponed to the detriment of the cardiac patient collective due to heightened protection requirements during the current pandemic. In the case of proven and not explicitly excluded SARS-CoV-2 infections, personal protective equipment should be used during the examination to protect the medical staff and other patients.

This pandemic represents a very challenging situation for healthcare workers and hospitals. Apart from adapting daily clinical routines to adequately meet patient needs while protecting healthcare workers, also concepts for echocardiography teaching and education require customization for patient and staff protection alike (18). As the status of this worldwide pandemic represents an ever-changing situation and knowledge regarding protective measures is expanding on a monthly basis we sought to provide a state of the art overview on current recommendations and novel concepts for cardiovascular imaging.

## Author Contributions

ML prepared the article. EP, CG, and BW provided additional informationen. KK revised the manuscript. AD and UH supervised the preparation of the article. All authors contributed to the article and approved the submitted version.

## Conflict of Interest

The authors declare that the research was conducted in the absence of any commercial or financial relationships that could be construed as a potential conflict of interest.

## References

[1] Cook TM. Personal protective equipment during the coronavirus disease (covid) 2019 pandemic - a narrative review. Anaesthesia. (2020) 75:920–7. 10.1111/anae.15071 32246849

[2] van Doremalen N Bushmaker T Morris DH Holbrook MG Gamble A Williamson BN. Aerosol and surface stability of sars-cov-2 as compared with sars-cov-1. N Engl J Med. (2020) 382:1564–7. 10.1056/NEJMc2004973 32182409 PMC7121658

[3] Dhama K Khan S Tiwari R Sircar S Bhat S Malik YS Coronavirus disease 2019-covid-19. Clin Microbiol Rev. (2020) 33:e00028–20. 10.1128/CMR.00028-20 32580969 PMC7405836

[4] Tran K Cimon K Severn M Pessoa-Silva CL Conly J. Aerosol generating procedures and risk of transmission of acute respiratory infections to healthcare workers: a systematic review. PLoS ONE. (2012) 7:e35797. 10.1371/journal.pone.0035797 22563403 PMC3338532

[5] Riediker M Tsai DH. Estimation of viral aerosol emissions from simulated individuals with asymptomatic to moderate coronavirus disease 2019. JAMA Netw Open. (2020) 3:e2013807. 10.1001/jamanetworkopen.2020.13807 32716517 PMC11875120

[6] Kirkpatrick JN Mitchell C Taub C Kort S Hung J Swaminathan M. Ase statement on protection of patients and echocardiography service providers during the 2019 novel coronavirus outbreak: endorsed by the american college of cardiology. J Am Soc Echocardiogr. (2020) 33:648–53. 10.1016/j.echo.2020.04.001 32503700 PMC7129086

[7] British Society of Echocardiography Clinical Guidance Regarding Provision of Echocardiography During the Covid-19 Pandemic. London (2020).

[8] Antonini-Canterin F Pepi M Monte IP Trocino G Barbieri A Barchitta A. Document addressed to cardiovascular echography operators at the time of covid-19: a document by the “società italiana di ecocardiografia e cardiovascular imaging” board 2019-2021. J Cardiovasc Echogr. (2020) 30:2–4. 10.4103/jcecho.jcecho_27_20 32766099 PMC7307620

[9] Seo Y Daimon M Yamada H Kagiyama N Ohta M Izumi C. Review of the efforts of the japanese society of echocardiography for coronavirus disease 2019 (covid-19) during the initial outbreak in japan. J Echocardiogr. (2020) 18:226–33. 10.1007/s12574-020-00487-5 32892279 PMC7474571

[10] Gupta R Das MK Mohanan PP Deb PK Parashar SK Chopra HK. Cardiological society of india document on safety measure during echo evaluation of cardiovascular disease in the time of covid-19. Indian Heart J. (2020) 72:145–50. 10.1016/j.ihj.2020.05.016 32768012 PMC7250084

[11] ESC Protecting Cardiologists During the Covid-19 Epidemic – Lessons From Wuhan, China. ESC. (2020).

[12] Skulstad H Cosyns B Popescu BA Galderisi M Salvo GD Donal E. Covid-19 pandemic and cardiac imaging: eacvi recommendations on precautions, indications, prioritization, and protection for patients and healthcare personnel. Eur Heart J Cardiovasc Imaging. (2020) 21:592–8. 10.1093/ehjci/jeaa072 32242891 PMC7184341

[13] Inciardi RM Lupi L Zaccone G Italia L Raffo M Tomasoni D. Cardiac involvement in a patient with coronavirus disease 2019 (covid-19). JAMA Cardiol. (2020) 5:819–24. 10.1001/jamacardio.2020.1096 32219357 PMC7364333

[14] Cameli M Pastore MC Henein M Aboumarie HS Mandoli GE D'Ascenzi F. Safe performance of echocardiography during the covid-19 pandemic: a practical guide. Rev Cardiovasc Med. (2020) 21:217–23. 10.31083/j.rcm.2020.02.90 32706210

[15] Viéitez Flórez JM Barrios Alonso V Fernández-Gofín C. The day after tomorrow: echocardiography laboratories after the covid-19 outbreak. Eur Heart J Cardiovasc Imaging. (2020) 21:1057. 10.1093/ehjci/jeaa207 32734283 PMC7454441

[16] Goldberg AB Kyung S Swearingen S Rao A. Expecting the unexpected: echo laboratory preparedness in the time of covid-19. Echocardiography. (2020) 37:1272–7. 10.1111/echo.14763 32657445 PMC7404746

[17] Augoustides JG. Perioperative echocardiography: key considerations during the coronavirus pandemic. J Cardiothorac Vasc Anesth. (2020) 34:1416–8. 10.1053/j.jvca.2020.03.046 32249075 PMC7138190

[18] Madrazo JA. New challenges and opportunities for echocardiographic education during the covid-19 pandemic: a call to focus on competency and pathology. J Am Soc Echocardiogr. (2020) 33:1048–9. 10.1016/j.echo.2020.05.011 32527626 PMC7260499

[19] McMahon SR De Francis G Schwartz S Duvall WL Arora B Silverman DI. Tablet-based limited echocardiography to reduce sonographer scan and decontamination time during the covid-19 pandemic. J Am Soc Echocardiogr. (2020) 33:895–9. 10.1016/j.echo.2020.05.005 32624089 PMC7211571

[20] Jenkins S Garg P. Prime time for handheld echocardiography in covid-19 pandemic. Clin Med. (2020) 20:e132. 10.7861/clinmed.Let.20.4.3 32675165 PMC7385783

[21] Jain A. Preventing contamination during transesophageal echocardiography in the face of the covid-19 pandemic. J Cardiothorac Vasc Anesth. (2020) 34:2849–51. 10.1053/j.jvca.2020.04.011 32362542 PMC7194988

[22] Senniappan K Damodaran S Kanchi M. Epicardial echocardiography-a plausible alternative cardiac imaging technique in covid-19 pandemic. J Cardiothorac Vasc Anesth. (2021) 35:684–6. 10.1053/j.jvca.2020.06.049 32654805 PMC7305729

[23] Sayburn A Covid-19: phe upgrades ppe advice for all patient contacts with risk of infection. BMJ. (2020) 369:m1391 10.1136/bmj.m1391 32245770

[24] Pradhan D Biswasroy P Kumar Naik P Ghosh G Rath G. A review of current interventions for covid-19 prevention. Arch Med Res. (2020) 51:363–74. 10.1016/j.arcmed.2020.04.020 32409144 PMC7190516

[25] Barker PCA Lewin MB Donofrio MT Altman CA Ensing GJ Arya B. Specific considerations for pediatric, fetal, and congenital heart disease patients and echocardiography service providers during the 2019 novel coronavirus outbreak: council on pediatric and congenital heart disease supplement to the statement of the american society of echocardiography: endorsed by the society of pediatric echocardiography and the fetal heart society. J Am Soc Echocardiogr. (2020) 33:658–65. 10.1016/j.echo.2020.04.005 32503702 PMC7144602

[26] Sirico D Castaldi B Ciliberti P Sabatino J Cazzoli I Secinaro A. Cardiac imaging in congenital heart disease during the coronavirus disease-2019 pandemic: recommendations from the working group on congenital heart disease of the italian society of cardiology. J Cardiovasc Med (Hagerstown). (2020) 21:467–71. 10.2459/JCM.0000000000000990 32487868 PMC7314347

[27] Sabatino J Ferrero P Chessa M Bianco F Ciliberti P Secinaro A. Covid-19 and congenital heart disease: results from a nationwide survey. J Clin Med. (2020) 9:1774. 10.3390/jcm9061774 32521643 PMC7355526

